# Relationship between serum bilirubin levels and cardiovascular disease

**DOI:** 10.1371/journal.pone.0193041

**Published:** 2018-02-15

**Authors:** Sunghwan Suh, Young Rak Cho, Mi Kyoung Park, Duk Kyu Kim, Nam H. Cho, Moon-Kyu Lee

**Affiliations:** 1 Department of Internal Medicine, Dong-A University Medical Center, Dong-A University College of Medicine, Busan, Republic of Korea; 2 Department of Preventive Medicine, Ajou University School of Medicine, Suwon, Republic of Korea; 3 Division of Endocrinology and Metabolism, Department of Medicine, Samsung Medical Center, Sungkyunkwan University School of Medicine, Seoul, Republic of Korea; German Diabetes Center, Leibniz Center for Diabetes Research at Heinrich Heine University Düsseldorf, GERMANY

## Abstract

We tested the hypothesis that higher levels of bilirubin, a bile pigment with antioxidant properties, are associated with a decreased risk of cardiovascular disease (CVD). This study analyzed data from the Korean Health and Genome Study to examine the association between serum total bilirubin (TB) on CVD and CVD death. Serum TB was measured in a total of 8,844 subjects (4,196 males and 4,648 females) and evaluated for the development of new onset CVD from 2001 to 2012 (mean 8.1 years of follow-up). During the follow-up period, 689 cases of incident CVD (7.8%) were identified, and the prevalence of metabolic syndrome (MetS) at baseline was 26.1%. The prevalence of MetS decreased across bilirubin tertile categories. In addition to MetS itself, individual components of MetS significantly decreased with increased bilirubin tertiles. Moreover, the incidence of CVD decreased across bilirubin tertile categories. The hazard ratios (HRs) for developing coronary heart disease (CHD, HR 0.769, 95% CI 0.655–1.000) and CVD death (HR 0.513, 95% CI 0.267–0.985) was significantly lower in the highest tertile group (> 0.63 mg/dL) in comparison to the lowest tertile group (< 0.44 mg/dL) after adjusting for all confounding variables. In the present longitudinal study, a significant negative relationship was demonstrated between baseline bilirubin levels and incident CHD and CVD death.

## Introduction

The role of inflammation in cardiovascular disease (CVD) is established. Oxidative stress plays an important role in atherosclerosis, which is a chronic inflammatory response to vascular endothelial injury caused by a variety of factors promoting inflammatory cell entry and activation [1]. The recognition of bilirubin as an important endogenous anti-inflammatory and antioxidant molecule has increased in recent decades. Bilirubin affects atherosclerosis by several inhibiting mechanisms, including low-density lipoprotein oxidation, vascular smooth muscle cell proliferation, and endothelial dysfunction [1]. Mildly elevated circulating bilirubin levels seems to represent a promising target for prevention and reduction of the prevalence of CVD and other oxidative-stress disorders, including type 2 diabetes mellitus (T2DM) and cancer [2]. Accordingly, the role of bilirubin as a biological predictor in the risk assessment of chronic disorders, with increasing worldwide prevalence, is of considerable medical economic importance. Indeed, CVD represent a main cause of mortality and burden of disease [3].

Recent meta-analysis has found an inverse association between total bilirubin levels and the risk of CVD, which is independent of established risk factors [4]. Thus, serum bilirubin level may be an independent marker for environmental and genetically determined CVD risk. The reported effects of bilirubin levels on an individual basis, however, have been inconsistent in the context of CVD. Increased bilirubin levels have been associated with greater protection against CVD in some studies [5, 6], whereas other research indicates that higher levels of bilirubin have increased or null associations with CVD [7, 8]. Because of the small sample sizes of previous studies, however, it is difficult to generalize the clinical implications to a wider population. In light of the ongoing debate on the potential value of total bilirubin levels in CVD risk prevention, comprehensive assessment of the association of baseline total bilirubin levels with risks for future CVD events and mortality using long-term observational evidence is demanding.

## Materials and methods

### Study population

The design and baseline characteristics of the Ansung-Ansan cohort study have been previously published [9]. Briefly, the study is an ongoing prospective, community-based cohort study that is part of the Korean Health and Genome Study (KHGS), which is a community-based epidemiological survey to investigate trends in diabetes mellitus (DM) and associated risk factors. The baseline examination was performed from 2001 to 2002, and biennial follow-up examinations were continued through 2012. As part of the biennial follow-up, researchers contacted all participants who did not attend the scheduled site visit by telephone or a door-to-door visit to encourage them to undergo follow-up examination. A total of 10,038 subjects aged 40–69 years (with the Ansung-Ansan cohort comprising 5,018 subjects from a farming community and 5,020 from an urban community, respectively) were enrolled in the KHGS. Throughout the study, the same trained researchers and instruments were used to collect data. Anthropometric parameters and blood pressure were measured by standard methods. Fasting plasma glucose, lipid profiles, and insulin were measured in a central laboratory. The homeostatic model assessment of insulin resistance (HOMA-IR), which is a method used to quantify insulin resistance (IR), was defined as [fasting insulin (μU/mL) × fasting glucose (mmol/L)] / 22.5. The disease and smoking status of participants was assessed by questionnaire. Current smokers were defined as those who had smoked at least one cigarette per day for at least the prior year. Serum bilirubin concentrations, aspartate aminotransferase (AST), and aminotransferase (ALT), were determined by a Hitachi 747 automated analyzer. Informed written consent was obtained from all participants. The study protocol was approved by the ethics committee of the Korean Center for Disease Control and the Institutional Review Board of Ajou University School of Medicine.

### Definition of the metabolic syndrome

Subjects were diagnosed with the metabolic syndrome (MetS) if they met at least three of the following revised criteria of the National Cholesterol Education Program Adult Treatment Panel III [10]: (1) abdominal obesity, (2) triglyceride levels of 150 mg/dL or greater or current use of lipid-lowering treatment, (3) high density lipoprotein cholesterol (HDL-C) levels of less than 40 mg/dL in males or less than 50 mg/dL in females, (4) blood pressure of 130/85 mm Hg or greater or current use of antihypertensive medications, or (5) fasting plasma glucose levels of 100 mg/dL or greater, previously diagnosed T2DM, or current use of oral antidiabetic agents or insulin. Abdominal obesity was defined as a waist circumference of ≥90 cm for males and ≥85 cm for females, which are the ethnically appropriate abdominal obesity criteria for Korean people as proposed by the Korean Society for the Study of Obesity [11].

### Determination of incident CVD

Coronary heart disease (CHD) was defined as definite myocardial infarction confirmed by electrocardiogram and/or enzyme changes or any angina diagnosis that required intervention after confirmation of coronary artery stenosis by coronary angiography. Stroke included cerebral infarction, hemorrhagic stroke, transient ischemic attack, and vertebrobasilar insufficiency as demonstrated by diagnostic work-ups, such as, computed tomography, magnetic resonance imaging studies and accompanying neurologic symptoms and/or signs. CVD was defined as the occurrence of CHD and/or stroke. Persons with medical events reported by the patient directly or found during routine follow-up examinations were asked to provide their medical records. Data on CVD events were also obtained from the reports of study participants. The CVD events reported by participants were corroborated by initial in-depth interviews, as well as interviews repeated at each biennial follow-up visit. Researchers contacted all participants who did not appear for follow-up examinations by telephone or a home visit, and all deaths were reported by the families of participants by way of these methods of contact. Death due to CHD or stroke was defined as a CVD death. Information about the death of participants, including the date, place, and cause, was obtained through interviews with the families of participants and reference to death certificates. Initial data were obtained from 10,038 subjects who participated in the KHGS. Among these subjects, 1,194 were excluded for the following reasons: (1) previous history of CVD (n = 230), (2) lack of follow-up examinations after baseline examination (n = 908), (3) serum TB levels of ≥ 2.0 mg/dL (n = 54), and (4) missing data (n = 2). After applying the above exclusion criteria, a total of 8,844 subjects were eligible for the study.

### Statistical analyses

Statistical analyses were carried out using SPSS version 22.0 (IBM Co., NY, USA). The results are expressed as subject number with percentage (%) and mean with standard deviation for each of the characteristics of the study participants. Subjects were categorized into three tertiles (Q1–Q3) according to baseline total bilirubin concentrations. One-way analysis of variance (ANOVA) and Pearson’s chi-square tests were used to analyze statistical differences in the characteristics of the study participants among tertiles. Trends between categorical variables were tested for statistical significance using chi-square tests for linear-by-linear association. The hazard ratios (HRs) for the association of serum total bilirubin with CVD events or mortality were estimated using a multivariable Cox proportional hazard model after adjusting for confounding variables. For all statistical analyses, a two-sided P<0.05 was considered statistically significant.

## Results

Subjects were categorized into tertiles based on baseline levels of serum total bilirubin. The mean level of serum TB was 0.59 mg/dL in the whole population. The clinical and biochemical characteristics of subjects according to these categories are shown in Table 1. Negative relationships were observed between the bilirubin tertiles and age, HbA1c, systolic blood pressure (SBP), diastolic blood pressure (DBP), high-sensitive C-reactive protein (hs-CRP), and HOMA–IR. In contrast, there was a positive relationship between bilirubin tertiles and the characteristic of being male, total cholesterol (TC) level, high-density lipoprotein cholesterol (HDL) level, low-density lipoprotein cholesterol (LDL) level, AST, ALT, and the prevalence of being a current smoker. Subjects in the highest bilirubin tertile group (Q3) had younger ages, were male subjects, had higher levels of fasting glucose, TC, HDL, LDL, AST, ALT, and a higher prevalence of being current smokers, as well as a lower waist circumference, HbA1c, SBP, and DBP in comparison with the lowest bilirubin tertile group (Q1). As the bilirubin tertile increased, the prevalence of MetS decreased (P<0.001). The prevalence of MetS decreased across bilirubin tertile categories (Table 2). In addition to MetS itself, individual components of the MetS significantly decreased as the bilirubin tertiles increased, with the exception of hypertriglyceridemia (Table 2). Among them, 689 subjects developed CVD events during the follow-up period (mean follow up of 8.1 years). There were 428 deaths, of which 84 were due to CVD during the follow-up period. The multivariable Cox regression model relating traditional risk factors to CVD events or mortality is given in Table 3 during the 8-year study period. There was a significant inverse relationship with bilirubin tertiles between CVD risk and mortality. The incidence of CVD and CVD death decreased across bilirubin tertile categories (P < 0.05). The HR of serum bilirubin as continuous variable (0.1~1.9 mg/dL) for incident CHD was 0.632 (95% CI 0.415–0.962, P = 0.032) after adjusting for potential confounders, including sex, age, body mass index (BMI), LDL, HbA1c, systolic blood pressure (SBP) and smoking status. The highest tertile was significantly associated with lower risks of CHD (HR 0.769, 95% CI 0.593–0.996, P = 0.047) and CVD death (HR 0.513, 95% CI 0.267–0.985, P = 0.045). The risk of incident CVD was marginally low in those with the highest bilirubin levels (HR 0.809, 95% CI 0.655–1.000, P = 0.050). However, there was no significant correlation of bilirubin tertiles with stroke or CHD death.

**Table 1:** Baseline characteristics of the study population.

N = 8,844	Serum total bilirubin tertile categories (mg//dL)			P value
	Q1 (< 0.44)	Q2 (0.44–0.63)	Q3 (>0.63)	
Number of subjects	2,951	2,942	2,951	
Age (years)	54.19 ± 9.00	52.12 ± 8.81	50.59 ± 8.55*	<0.001
Male	902 (30.6)	1,364 (46.4)	1,930 (65.4)*	<0.001
BMI (kg/m^2^)	24.52 ± 3.29	24.63 ± 3.18	24.53 ± 2.93	0.261
Waist (cm)	83.21 ± 9.13	82.52 ± 8.83	82.43 ± 8.42*	0.116
Fasting glucose (mg/dL)	86.30 ± 16.83	89.45 ± 23.11	90.82 ± 23.99*	<0.001
HbA1c (%)	5.84 ± 0.88	5.82 ± 0.98	5.73 ± 0.92*	<0.001
SBP (mmHg)	119.62 ± 18.52	117.48 ± 18.00	115.41 ± 17.47*	0.013
DBP (mmHg)	75.48 ± 11.11	74.96 ± 11.48	74.72 ± 17.47*	0.036
TC (mg/dL)	189.58 ± 35.14	195.30 ± 36.15	195.38 ± 36.53*	<0.001
TG (mg/dL)	157.47 ± 91.25	160.16 ± 107.26	157.78 ± 110.64	0.142
HDL (mg/dL)	45.82 ± 10.61	46.38 ± 10.95	46.71 ± 11.02*	0.003
LDL (mg/dL)	114.76 ± 32.97	120.59 ± 36.33	120.86 ± 36.50*	<0.001
hs-CRP (mg/dL)	0.27 ± 0.70	0.23 ± 0.47	0.21 ± 0.37*	<0.001
HOMA-IR	1.84 ± 1.33	1.70 ± 1.19	1.57 ± 1.40*	<0.001
Total serum bilirubin (mg/dL)	0.33 ± 0.07	0.53 ± 0.06	0.89 ± 0.25*	<0.001
AST (mg/dL)	26.13 ± 11.44	27.72 ± 16.70	30.71 ± 20.08*	<0.001
ALT (mg/dL)	24.20 ± 16.88	26.85 ± 21.98	29.42 ± 26.01*	<0.001
Current Smoker	668 (22.7)	769 (26.2)	800 (27.1)*	<0.001

Data are expressed as mean ± SD or number of subjects (%).

*Significantly different from the Q1 group (*P*<0.05).

BMI, body mass index; HbA1c, glycated hemoglobin; SBP, systolic blood pressure; DBP, diastolic blood pressure; TC, total cholesterol; TG, triglycerides; HDL, high-density lipoprotein cholesterol; LDL, low-density lipoprotein cholesterol; hs-CRP, high-sensitive C-reactive protein; HOMA-IR, homeostatic model assessment of insulin resistance; AST, aspartate aminotransferase; ALT, alanine aminotransferase.

**Table 2:** Metabolic syndrome at baseline according to serum total bilirubin tertiles.

N = 8,844	Serum total bilirubin tertile categories (mg//dL)			P value
	Q1 (< 0.44)	Q2 (0.44–0.63)	Q3 (>0.63)	
Number of subjects	2,951	2,942	2,951	
MetS (n = 2,307)	894 (30.3)	775 (26.3)	638 (21.6)	<0.001
MetS components	1.8 ± 1.3	1.7 ± 1.3	1.5 ± 1.2	<0.001
Abdominal obesity (n = 2,627)	1,069 (36.2)	849 (28.9)	709 (24.0)	<0.001
Hypertension (n = 2,724)	978 (33.1)	925 (31.4)	821 (27.8)	<0.001
Low HDL (n = 4,362)	1,690 (57.3)	1,452 (49.4)	1,220 (41.3)	<0.001
High TG (n = 3,557)	1,205 (40.8)	1,175 (39.9)	1,177 (39.9)	0.706
Hyperglycemia (n = 1,621)	499 (16.9)	571 (19.4)	551 (18.7)	0.039

Data are expressed as number of subjects (%). Subjects were diagnosed with the metabolic syndrome components if they met at least three of the following revised criteria of the National Cholesterol Education Program Adult Treatment Panel III [10]. Abdominal obesity was defined as criteria for Korean people as proposed by the Korean Society for the Study of Obesity [11]. MetS, metabolic syndrome; TG, triglycerides; HDL, high density lipoprotein cholesterol.

**Table 3:** Risk of cardiovascular disease and mortality based on serum total bilirubin tertiles during follow-up.

N = 8,844	Serum total bilirubin tertile categories (mg//dL)			P value
	Q1 (< 0.44)	Q2 (0.44–0.63)	Q3 (>0.63)	
Number of subjects	2,951	2,942	2,951	<0.001
* *CVD (n = 689)	274 (9.3)	232 (7.9)	183 (6.2)	<0.001
* *CHD (n = 444)	181 (6.1)	150 (5.1)	113 (3.8)	<0.001
* *Stroke (n = 270)	104 (3.5)	84 (2.9)	82 (2.8)	0.187
* *CVD death (n = 84)	37 (1.3)	33 (1.1)	14 (0.5)	0.004
* *CHD death (n = 50)	18 (0.6)	23 (0.8)	9 (0.3)	0.047
Adjusted hazard ratio (95% CI)				
Multivariate model 1				
* *CVD (n = 689)	1	0.953 (0.789–1.151)	0.803 (0.652–0.988)*	
* *CHD (n = 444)	1	0.939 (0.747–1.180)	0.765 (0.593–0.987)*	
* *Stroke (n = 270)	1	0.903 (0.670–1.218)	0.949 (0.695–1.296)	
* *CVD death (n = 84)	1	1.003 (0.619–1.626)	0.448 (0.236–0.849)*	
* *CHD death (n = 50)	1	1.521 (0.810–2.856)	0.660 (0.288–1.514)	
Multivariate model 2				
* *CVD (n = 689)	1	0.937 (0.775–1.136)	0.809 (0.655–1.000)	
* *CHD (n = 444)	1	0.916 (0.727–1.154)	0.769 (0.593–0.996)*	
* *Stroke (n = 270)	1	0.903 (0.668–1.221)	0.969 (0.706–1.331)	
* *CVD death (n = 84)	1	1.083 (0.661–1.774)	0.513 (0.267–0.985)*	
* *CHD death (n = 50)	1	1.562 (0.817–2.986)	0.728 (0.312–1.699)	

Data are expressed as number of patients (%) or hazard ratio (95% confidence interval) of patients (%).CVD, cardiovascular disease; CHD, coronary heart disease. Model 1: adjusted for age and sex. Model 2: adjusted for sex, age, BMI, LDL-C, HbA1c, SBP and smoking. Model 3: adjusted for sex, age, BMI, LDL-C, HbA1c, SBP smoking, HOMA-IR and hsCRP.

* p < 0.05

## Discussion

The present longitudinal study demonstrates a significant negative relationship between baseline bilirubin levels within the physiologic range and incident CHD/CVD death, thereby suggesting that highest tertile of serum bilirubin may play a protective role against incident CHD and CVD death. Furthermore, serum bilirubin is negatively associated with MetS and MetS components. These findings suggest that elevation of bilirubin levels within the normal range are a favorable condition from a metabolic standpoint.

These data also suggest that baseline circulating total bilirubin levels are significantly and inversely associated with CHD outcomes in the general population, which is consistent with the findings of a previous cohort study [4, 12]. Plausible biological mechanisms by which higher serum total bilirubin contributes to reduced CVD risk include the antioxidant actions of serum bilirubin (through the inhibition of low-density lipoprotein oxidation), anti-inflammatory effects, anti-atherogenic properties, or, as more recently reported, pathways associated with vascular structure and reactivity [2, 12, 13]. In-vitro studies have shown that bilirubin is a powerful endogenous antioxidant, which scavenges peroxyl and hydroxyl radicals [8, 12]. In line with previous data that suggests the possible involvement of oxidative stress in the pathogenesis of IR and MetS [14], the antioxidant capacity of bilirubin might be involved in preventing incident MetS. Another mechanism is potentially related to the anti-inflammatory effects of bilirubin. Considering that chronic inflammation leads to IR, which is a central feature of MetS [15], it seems plausible that the protective roles of serum bilirubin against inflammation and IR might also prevent incident MetS. The work of Oda et al. found that decreased serum bilirubin is a marker of oxidative stress [16], which is closely related to inflammation and endothelial dysfunction. Both inflammation and endothelial dysfunction are thought to be underlying mechanisms of MetS [16]. In this study, both markers of inflammation and IR (hs-CRP and HOMA-IR) are negatively associated with serum total bilirubin levels. In addition, serum bilirubin is negatively associated with MetS and MetS components. Smoking is positively associated with serum bilirubin levels in our study. These findings are consistent with those from previous studies [17, 18]. Smoking generates many oxidants and free radicals, the presence of which is closely related to serum bilirubin levels [19, 20]. For these reasons, a pro-oxidant/antioxidant imbalance in the blood and tissue may be induced by smoking. In addition, the high prevalence of smoking in the Q3 group might be due to the high number of male subjects in the group. However, our results remain significant when smoking status is adjusted for the analysis. Several prospective studies did not found protective effect of bilirubin on CVD [21]. However, these studies were limited by its small sample size or patients with high risk profile for CVD.

At baseline values, inverse correlations were found between serum bilirubin concentration and abdominal obesity, hypertension, DM, and IR, which are similar results to the findings of previous prospective studies [22]. Because metabolic disorder and IR are also known to increase the risk for CVD [23], it is possible to speculate that the association between bilirubin levels and the risk of future CVD observed herein may be caused by these metabolic effects of bilirubin. Additional to the mechanisms already known, a significant negative relationship is observed in this study between bilirubin and individual components of MetS. MetS is a constellation of interrelated metabolic risk factors that appear to promote the development of CVD [24]. We have already shown that MetS and some components of them are significantly associated with the risk of developing CVD [25]. Several recent studies also reported a negative association between bilirubin and MetS [16, 18, 26]. However, most of these previous studies are cross-sectional in design and deal mostly with routine medical check-ups of subjects. Moreover, the existing studies do not fully elucidate the longitudinal association between bilirubin and the risk of developing CVD in the Asian cohort.

For the first time in the Asian cohort, a significant trend was found between bilirubin tertiles and CVD/CHD mortality. In addition, we found a significant association of bilirubin concentration with MetS. To help characterize and more reliably quantify the nature and magnitude of these associations, we provided a detailed assessment of the association of total bilirubin levels with the risk of CVD in a large cohort of 8,844 participants free from CVD at entry. Our study is the first to show the association between serum bilirubin levels and the MetS-CHD-CVD death continuum. However, we did not identify an association in stroke and CHD death. A previous Belgian study also failed to show the association [8]. The number of events may have been too small, and therefore the power too low, to detect associations with stroke and CHD death in this population. Serum bilirubin and its use for CVD risk prevention has been proposed because serum bilirubin is a simple, standardized, cost effective, scalable biomarker, and with a potential role in the cause of CVD [27]. The overall evidence from this study also supports the association between circulating total bilirubin levels within the physiologic range and CVD events in the general population. While direct intravenous administration of bilirubin or its soluble precursor, biliverdin, has been shown to benefit rats exposed to endotoxins [28], the use of bilirubin in humans requires further preclinical development.

The lack of total bilirubin fractionation (indirect and direct) has restricted the ability to evaluate whether direct or indirect hyperbilirubinemia is associated with CVD risk. However, there is a strong correlation between total bilirubin and unconjugated bilirubin, as well as between total bilirubin and conjugated direct bilirubin in healthy subjects [29]. We do not have detailed data on pathologic jaundice (e.g. hepatitis, liver cirrhosis, herbs or alternative medicines, etc.). Therefore, we tried to eliminate these potentials by excluding those with serum TB levels of ≥ 2.0. Nevertheless, this study prospectively showed the effects of bilirubin levels on CHD risk and CVD mortality in a large community-based cohort, which has been previously conducted in only a very limited number of studies. Low bilirubin levels can be indicative of decreased heme oxygenase activity (a powerful antioxidant) or could be indicative of high oxidative stress in patients leading to consumption of the natural antioxidants including bilirubin. Hence, there is possibility that lower levels of bilirubin are perhaps not the causal factor for CVD but may indicate patients at an increased risk of developing CVD [21]. Moreover, further large scale long-term study will be required to confirm our results regarding inflammation and IR in the future.

In the present longitudinal study, a significant negative relationship was demonstrated between baseline bilirubin levels and incident CHD and CVD death.
